# Bypass and Ligation of Right Subclavian Artery Aneurysm in a Patient with Marfan’s Syndrome via Reoperative Partial Upper Median Sternotomy

**DOI:** 10.21470/1678-9741-2023-0300

**Published:** 2024-10-14

**Authors:** Kevin R. An, Lamia Harik, Talal Alzghari, Roberto Perezgrovas-Olaria, Giovanni Jr. Soletti, Arnaldo Dimagli, Gianmarco Cancelli, Mario F.L. Gaudino, Sharif H. Ellozy, Christopher Lau

**Affiliations:** 1 Department of Cardiothoracic Surgery, New York-Presbyterian Hospital/Weill Cornell Medical Center, New York, New York, United States of America; 2 Division of Vascular Surgery, Department of Surgery, New York-Presbyterian Hospital/Weill Cornell Medical Center, New York, New York, United States of America

**Keywords:** Marfan Syndrome, Subclavian Artery, Aneurysm, Thromboembolism.

## Abstract

Subclavian artery aneurysms are rare and can result in thromboembolism or rupture. We present the case of a 41-year-old man with a history of Marfan’s syndrome and multiple previous operations, who presented with an enlarging asymptomatic 5.2 cm right subclavian artery aneurysm and was successfully treated with a hybrid surgical operation.

## INTRODUCTION

**Table:** 

Abbreviations, Acronyms & Symbols	
CTA	= Computed tomography angiography

First ligated by Abraham Colles in 1811, subclavian artery aneurysms are rare and estimated to comprise 0.5% of all peripheral artery aneurysms^[1^,^2]^. These aneurysms are associated with atherosclerosis, trauma, thoracic outlet syndrome, Marfan’s syndrome, and other connective tissue disorders^[3]^. We present the case of an enlarging asymptomatic 5.2 cm right subclavian artery aneurysm in a patient with Marfan’s syndrome and multiple previous operations who was successfully treated with a hybrid surgical operation.

## CASE PRESENTATION

A 41-year-old man who was monitored by serial follow-up imaging for his previous aortic operations was found to have an enlarging 5.2 cm right subclavian artery aneurysm. He was asymptomatic, with equal and palpable distal pulses bilaterally, and in no acute distress. The patient had a history and family history of Marfan’s syndrome (mother, maternal uncle, and maternal grandfather). In 2000, he underwent a Yacoub aortic valve-sparing root repair. Ten years later, he underwent a redo-sternotomy with bioprosthetic aortic valve replacement due to aortic valve endocarditis from *viridans* group streptococci. In 2011, he developed an uncomplicated type B aortic dissection which was initially medically managed, but later developed aneurysmal degeneration, requiring extent one and three thoracoabdominal aneurysm repairs in 2012 and 2015, respectively. During his previous operations, the right subclavian and axillary arteries were neither cannulated nor instrumented.

**Fig. 1:**
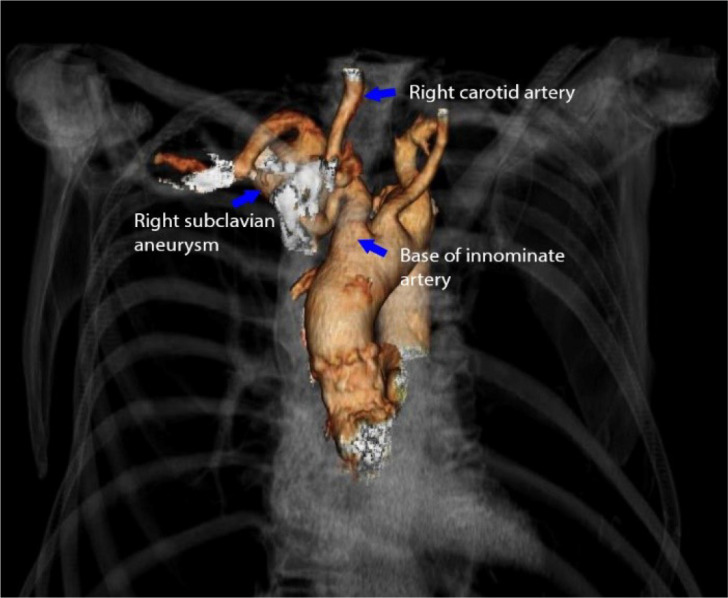
Computed tomography angiography with three-dimensional reconstruction of the right subclavian artery aneurysm demonstrating the tortuosity of the right subclavian aneurysm along with the base of the innominate artery and the right carotid artery (blue arrows)

His preoperative computed tomography angiography (CTA) scan showed a 5.2 cm right subclavian artery aneurysm with posterior wall thrombus (axial view, Video 1). The patient’s most recent CTA scan performed five years before showed a small 1.7 cm right subclavian artery aneurysm. A three-dimensional reconstruction of the CTA scan demonstrated the proximal location and tortuosity of the aneurysm (Figure 1).

**Video 1:**
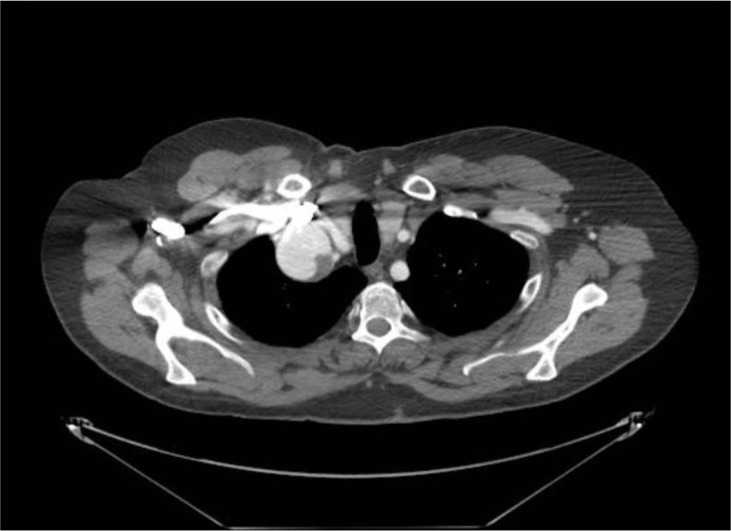
Axial computed tomography angiography demonstrating the size and location of the 5.2 cm right subclavian artery aneurysm Link: https://youtu.be/zVg2FdeA008

**Fig. 2:**
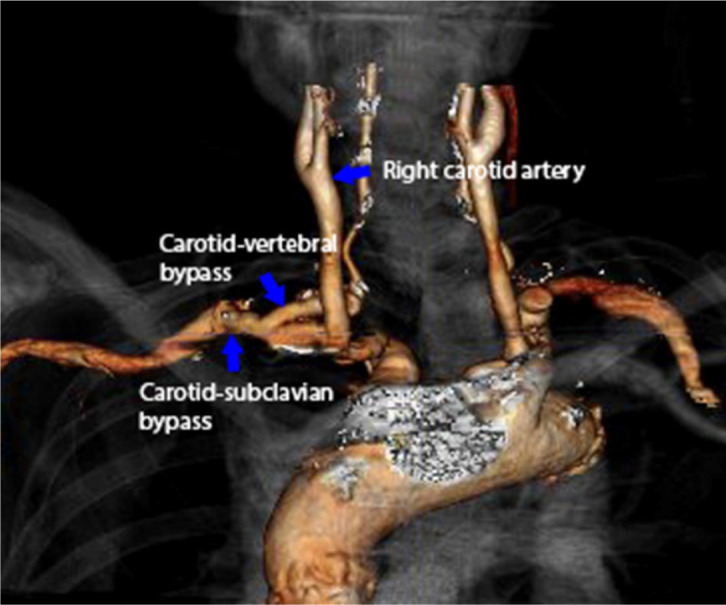
Postoperative computed tomography angiography with three-dimensional reconstruction of the right subclavian artery aneurysm. The right subclavian artery aneurysm has been repaired with carotid-subclavian and carotid-vertebral bypasses, ligation of the proximal subclavian artery, and coil embolization of the thyrocervical trunk.

Given his history of Marfan’s syndrome and type B aortic dissection, as well as serial growth on repeat imaging, we decided to pursue elective repair of his subclavian artery aneurysm. The patient was evaluated by a multidisciplinary team, including cardiothoracic surgeon and vascular surgeon, to explore management options. An endovascular approach was considered; however, given the size of the proximal innominate artery and the tortuosity of the common carotid artery, a device that would provide a suitable long-term repair could not be found (Figure 2)^[4]^. Due to his Marfan’s syndrome and young age, along with the long-term risks of stent migration and in-stent restenosis, it was felt that a hybrid approach involving a carotid-subclavian and carotid-vertebral bypass with surgical ligation of the proximal subclavian artery would be more appropriate.

**Fig. 3:**
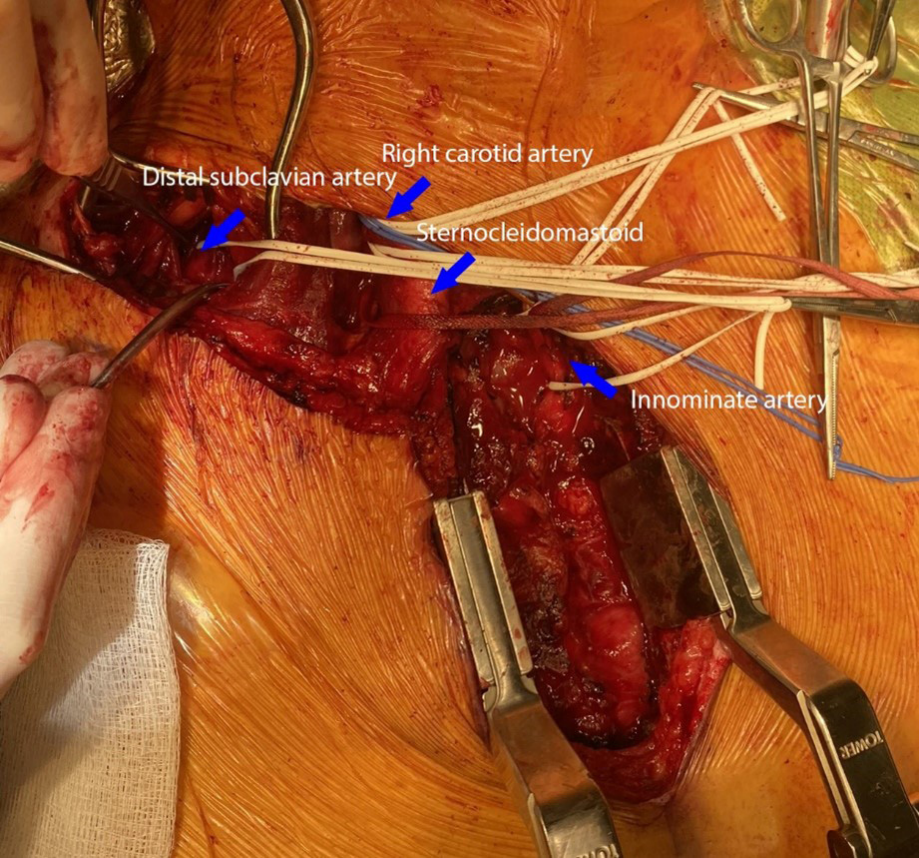
Operative view of the partial median sternotomy and supraclavicular incision. An operative view with the sternal retractor near the bottom right and the patient’s neck near the top. The innominate artery, sternocleidomastoid, right carotid artery, and distal subclavian artery are encircled with loops (blue arrows).

**Fig. 4:**
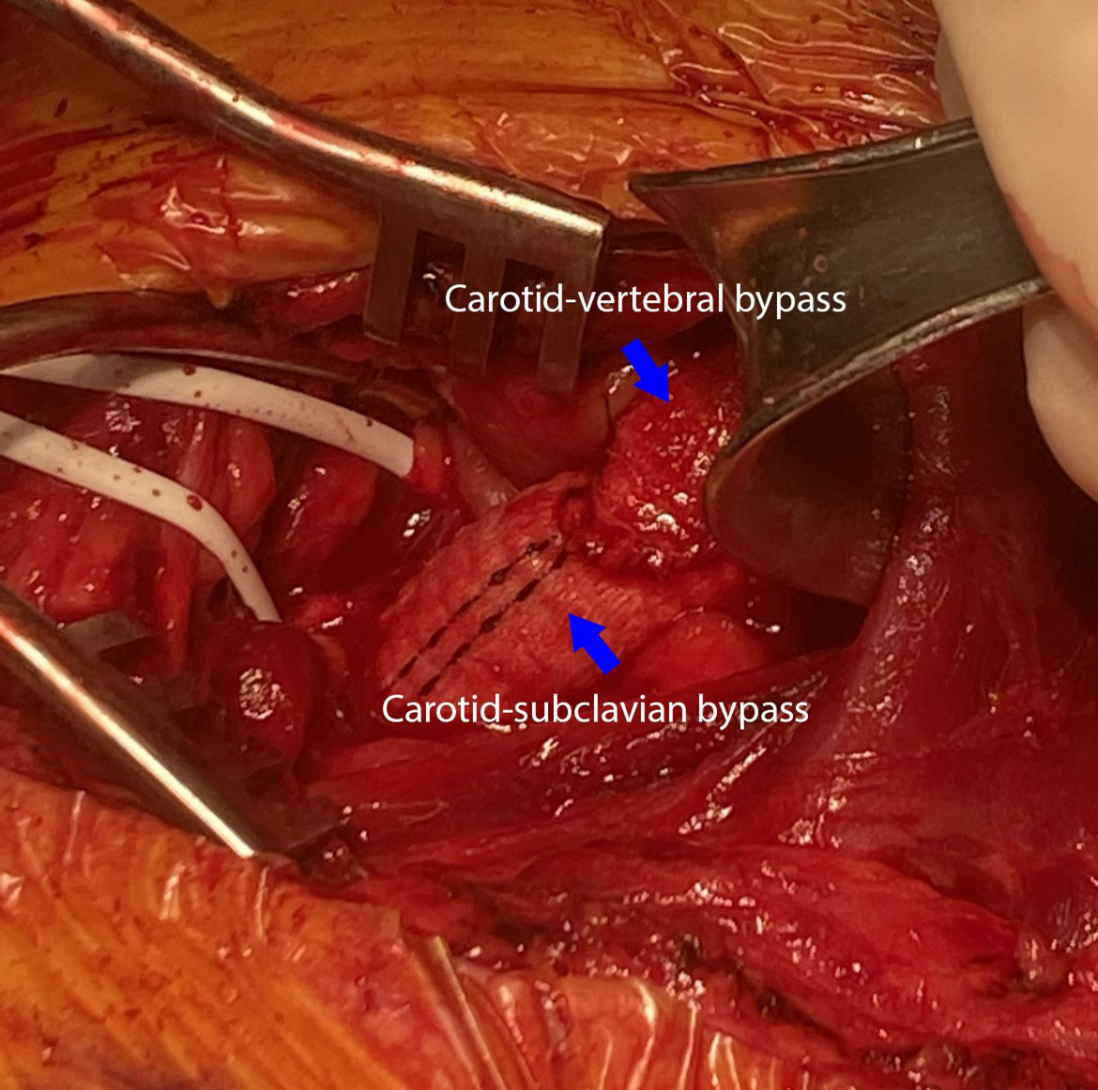
Operative view of the carotid-subclavian and carotid-vertebral artery bypasses. An operative view demonstrating the carotid-vertebral bypass with 6 mm Dacron graft and the carotid-subclavian bypass with 10 mm Dacron graft sewn end-to-side.

We performed a redo-partial upper median sternotomy and dissected free the innominate artery and origins of the right subclavian artery and common carotid artery (Figure 3). The right internal mammary artery was identified and ligated. The incision was extended into a right supraclavicular incision, and the carotid artery and large vertebral artery were encircled. The distal subclavian artery was identified in the supraclavicular space. Heparin was administered and the right vertebral artery was bypassed with a 6 mm Dacron graft in end-to-side fashion. The right carotid-subclavian bypass was performed with a 10 mm Dacron graft (Figure 4). The right subclavian artery and right vertebral artery were stapled and suture ligated, respectively, at their origins. An angiogram of the carotid-subclavian bypass allowed the identification of the thyrocervical trunk, which was coil embolized. The patient recovered well and was discharged from hospital on postoperative day three with no complications.

At three-month follow-up, he had recovered from his surgery and remained asymptomatic. His postoperative CTA scan demonstrated a thrombosed subclavian aneurysm sac (Video 2). His postoperative carotid and upper extremity arterial duplex revealed antegrade vertebral artery flow bilaterally and a patent right carotid-subclavian and carotid-vertebral bypass graft.

**Video 2:**
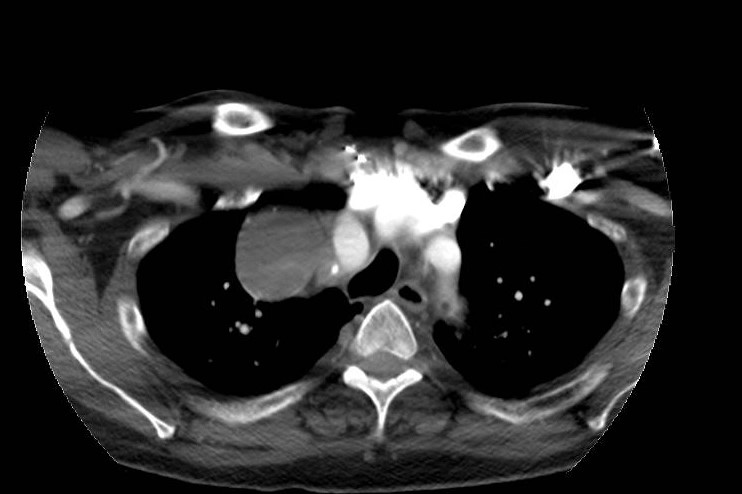
Operative view of the carotid-subclavian and carotid-vertebral artery bypasses. An operative view demonstrating the carotid-vertebral bypass with 6 mm Dacron graft and the carotid-subclavian bypass with 10 mm Dacron graft sewn end-to-side.

*Link: https://youtu.be/HwMEjSxW8Nc*

## DISCUSSION

When present in Marfan’s patients, subclavian artery aneurysms are often associated with multiple aneurysms and dissections, similar to our case, and close imaging surveillance is recommended^[5]^. When left untreated, they can lead to thrombosis, embolism, or rupture. Approximately half of all subclavian artery aneurysms present with symptoms, including shoulder pain, non-specific chest pain, pulsating mass, local compression, numbness, paresthesia, and hemoptysis^[3]^. The size cutoff for repair of subclavian artery aneurysms is unclear due to the rarity of such aneurysms, although a cutoff of 3 cm has been suggested^[6]^. This cutoff is supported by a review of 147 supra-aortic aneurysms, including 30 right subclavian artery aneurysms, which showed that small supra-aortic aneurysms, with a mean diameter of 1.97 ± 0.46 cm, grew slowly at a rate of 0.04 ± 0.099 cm per year, with no observed ruptures^[7]^. The approach to repairing such aneurysms varies and can include a combination of surgical and endovascular approaches, including carotid-subclavian, carotid-axillary, carotid-brachial, and/or carotid-vertebral bypass, interposition grafting, arterial ligation, subclavian artery stenting, innominate to carotid artery stenting, vascular plugs, and/or endovascular coiling^[5^,^6]^.

In our case, we considered an endovascular approach due to the difficulty of accessing the proximal right subclavian artery and the patient’s history of multiple previous operations. However, we could not find a suitable device due to the size of the innominate artery and tortuosity of the common carotid artery. Additionally, an endovascular approach would require stent placement in the distal common carotid artery, which has unknown long-term patency, particularly important given the patient’s young age. These issues are compounded by the increased risks associated with stent placement in patients with Marfan’s syndrome. A previous case reported a hybrid approach involving a carotid-subclavian and carotid-vertebral bypass with ligation of the subclavian artery distal to the aneurysm and covered stent of the innominate to carotid artery, which could be an alternative approach in cases where stenting of the innominate and carotid arteries is feasible^[8]^.

Cases of right subclavian artery aneurysm in Marfan’s patients are particularly rare, with two cases reported to date^[5^,^9]^. Notably, both cases involved patients with multiple arterial aneurysms and aortic dissection, which required multiple operations to repair. This suggests that right subclavian artery aneurysms may be associated with particularly severe cases of Marfan’s syndrome and demonstrates the importance of close long-term imaging surveillance in this patient population.

## CONCLUSION

We describe a rare case of a massive right subclavian artery aneurysm in a patient with Marfan’s syndrome and multiple previous operations. We provide a successful management approach for the aneurysm, involving carotid-subclavian and carotid-vertebral bypass grafts along with ligation of the aneurysm and endovascular coiling of the thyrocervical trunk in our patient.
